# How to be pregnant as a woman with ADHD and have children with ADHD

**DOI:** 10.1192/j.eurpsy.2026.11937

**Published:** 2026-07-31

**Authors:** A. Philipsen

**Affiliations:** Privatpsykiaterne ved Anne Philipsen, Soroe, Denmark

## Abstract

**Introduction:**

How to be pregnant as a woman with ADHD and have children with ADHD.

**Objectives:**

Women with ADHD are often facing more problems during pregnancy, after birth and during the child’s childhood than women without ADHD.

**Methods:**

We know that women are vulnerable during fall off estrogen and that they are at a higher risk of getting a post natal depression.

If the child has ADHD it is even more difficult for the women with ADHD.

**Results:**

Women with ADHD are vulnerable during pregnancy, not only because of the hormones, but also if the medication is taken from them or paused.

**Conclusions:**

Guidelines for treating women with ADHD during pregnancy are not really available yet. We have many clinical cases that can help us to handle ADHD-women during pregnancy.

**Disclosure of Interest:**

None Declared

